# Vitamin D status, vitamin D receptor gene polymorphism, and haplotype in patients with cutaneous leishmaniasis: Correlation with susceptibility and parasite load index

**DOI:** 10.1371/journal.pntd.0011393

**Published:** 2023-06-15

**Authors:** Doaa A. Salem, Mohammad A. Alghamdi, Hasan S. AL-Ghamdi, Bakheet A. Alghamdi, Ayman Zaki Elsayed Elsamanoudi, Abdulkarim Hasan

**Affiliations:** 1 Department of Medical Parasitology, Mansoura University, Faculty of Medicine, Mansoura, Egypt; 2 Department of Internal Medicine (Dermatology), Al-Baha University, Faculty of Medicine, Al-Baha, Saudi Arabia; 3 Department of Emergency Medicine, King Saud Medical City, Riyadh, Saudi Arabia; 4 Department of Clinical-Biochemistry, King Abdulaziz University, Faculty of Medicine, Jeddah, Saudi Arabia; 5 Department of Pathology, Al-Azhar University, Faculty of Medicine, Cairo, Egypt; CSIR-Indian Institute of Chemical Biology, INDIA

## Abstract

**Background:**

CL endemicity was reported worldwide including in Saudi Arabia, imposing a major challenge on the health authorities. Vitamin D and its receptor (VDR) are key modulators of the immune response where the VDR is expressed. A remarkable lack of data exists in humans about the contribution of vitamin D and polymorphisms of the VDR gene in protozoan infections, especially cutaneous leishmaniasis (CL).

**Objective:**

This is the first work conducted to assess the relationship between vitamin D status, polymorphisms of the VDR gene (BsmI, ApaI, TaqI, and FokI), and VDR haplotype with parasite tissue load and susceptibility to CL.

**Methods:**

Fifty-two patients with confirmed CL (21 patients receiving vitamin D medication and 31 patients not receiving it) and 46 control subjects participated in this cross-sectional investigation. VDR genotyping was determined by restriction fragment length polymorphism analysis. Serum levels of 25-OH vitamin D were assessed using the ELISA method in all participants. The skin biopsy quantified the parasite load based on the Ridley parasitic index.

**Results:**

The mean serum level of 25-OH vitamin D in CL patients who were not receiving vitamin D therapy was significantly lower compared to CL patients on vitamin D therapy and controls (p <0.001 for both) and CL patients with no history of vitamin D therapy had a significantly higher frequency of vitamin D deficiency compared to CL patients on vitamin D therapy and controls (p < 0.05). Compared to CL patients with no history of vitamin D therapy, CL patients receiving vitamin D therapy had a significantly lower mean size of the lesion and RPI (p = 0.02, .03 respectively). The frequency of genotype “aa” and its “a” allele in ApaI SNP of VDR was significantly lower in CL patients compared to controls (p = 0.006 and 0.03 respectively). However, patients with CL had a considerably greater frequency of the "A" allele than the controls (p = 0.03), suggesting its role in CL susceptibility. There was no statistically significant difference between the two groups in the genotype and allele frequency distributions of BsmI, TaqI, and FokI (p > 0.05). When compared to controls, CL cases had a considerably greater frequency of the "B-A-T-F" haplotype (p = 0.04), and a significantly lower frequency of the "B-a-T-F" haplotype (p = 0.01) suggesting that these haplotypes may have the potential susceptibility or protection against CL respectively. The "Aa" genotype in ApaI SNP of VDR had considerably lower levels of vitamin D with higher parasite load compared to the “AA” and: aa” genotypes (p = 0.02,0.02 respectively). A significant negative correlation was found between the parasite load and 25-OH vitamin D levels (r^2^ = -0.53, p< 0.001).

**Conclusions:**

According to these findings, vitamin D levels and "ApaI" VDR gene polymorphisms could affect the parasite load and susceptibility to infection, whereas BsmI, FokI, and TaqI polymorphisms did not. Correction of vitamin D levels may aid in CL management.

## Author summary

Cutaneous leishmaniasis (CL) is the most prevalent type of leishmaniasis with varied clinical presentations. It is caused by a group of protozoan intracellular parasites belonging to the genus Leishmania and transmitted through the bite of blood-sucking female sandfly species. few studies have examined the relationship between vitamin D and the VDR gene polymorphism and protozoal infection including leishmaniasis. Therefore, in this study, we aimed to investigate for the first time the possible association of VDR gene polymorphisms (BsmI, ApaI, TaqI, and FokI genotypes) with parasite load and susceptibility to CL and attempted to ascertain such an a ociation with serum levels of 25(OH) vitamin D in a trial to improve treatment strategies for CL.the results revealed that 25-OH vitamin D level was significantly lower among patients with CL compared to controls. The frequency of genotype “aa” and its “a” allele in ApaI SNP of VDR was significantly lower in CL patients compared to controls. However, the “A” allele frequency was significantly higher in patients with CL compared to controls, suggesting its role in CL susceptibility. The distribution of genotype and allele frequencies of BsmI, TaqI, and FokI were non-significantly different 86 between both groups. The parasite load was significantly negatively correlated with 25-OH vitamin D levels. Correction of vitamin D levels may aid in CL management.

## Introduction

The protozoan parasitic disease known as leishmaniasis is a vector-borne infection caused by obligate intracellular parasites of the leishmania species. The most prevalent type of leishmaniasis is cutaneous leishmaniasis (CL), with varied clinical presentations. Though CL is self-healing, the resulting scarring can be extremely disfiguring, causing psychological and social problems for those affected distress [1,2].

Over 90% of all CL cases have been documented in Afghanistan, Pakistan, Syria, Saudi Arabia, Algeria, and Iran, and it is considered a neglected tropical disease. CL is endemic in many parts of Saudi Arabia and continues to be a significant public health issue [3,4].

The active form of vitamin D, 1,25 dihydroxy-vitamin D3, regulates calcium and bone metabolism as well as the immunological function by interacting with the vitamin D receptor (VDR) on monocytes, macrophages, and activated lymphocytes [5].

According to the currently available literature, the population of Saudi Arabia has a 60% prevalence of hypovitaminosis D [6]. On the other hand, Al-Daghri et al. reported that the prevalence of vitamin D deficiency has decreased from 87.1% to 64.7% in people aged 18–40 years and from 86.2% to 45.7% in people aged > 40 years [7].

The VDR is a nuclear receptor superfamily that is encoded by a gene located on chromosome 12, with 8 coding and 3 non-coding regions. When this molecule binds to the active form of vitamin D, it dimerizes with the retinoid X receptor (RXR), which controls specific gene transcription [8].

The VDR locus contains five polymorphisms: the exon 2 initiation codon polymorphism, detected by FokI, [9], a cluster of polymorphisms in the 3′ ends of the VDR gene, defined by the restriction enzymes BsmI, ApaI, and TaqI [10], and the polyadenylase polymorphism further down the VDR 3′-untranslated region [11].

The VDR gene polymorphisms ApaI, BsmI, TaqI, and FokI have been studied in many infections including toxoplasmosis, tuberculosis, and *T*. *cruzi*. The changed VDR cannot bind to vitamin D and might no longer support macrophages in preventing intracellular bacterial development [12–14].

It is unclear how vitamin D and its receptor activity could be related to protozoal infections. To our knowledge, little research has studied the link between vitamin D and the VDR gene polymorphism and leishmaniasis; however, no research has linked these factors to the parasite load found in skin biopsies. Therefore, this is the first study designed to investigate the possible association of polymorphisms of the VDR gene (BsmI, ApaI, TaqI, and FokI genotypes) with parasite load and susceptibility to CL and tried to confirm such a link with serum levels of 25-OH vitamin D in attempted to improve CL treatment options.

## Patients and methods

### Ethics statement

This study protocol was approved by the Research Ethics Committee of Al-Bah College of Medicine (Approval No. REC/MED/BU.FM/2022/36). Following oral and written information about the trial, all participants in the study gave their written informed consent.

### Study design

The current study was a cross-section study. It is conducted between February 2020 and January 2023 in Al-Baha region, Saudi Arabia. Al-Baha is located in the Hejazi region of Saudi Arabia, west of the Kingdom of Saudi Arabia. Its climate is moderate in summer and cold in winter due to its elevation of 2,500 meters above sea level.

### Study population

The present study was carried out on 52 patients with confirmed cutaneous leishmaniasis (31 patients with no history of vitamin D therapy and 21 on vitamin D therapy) attendants of Dermatology Outpatient Clinics of Prince Mishari Bin Saud Hospital at Baljurashi, Al-Baha region, Saudi Arabia. Cutaneous leishmaniasis was diagnosed clinically by observing the lesion, along with a microscopic examination of the skin smear or biopsy to assess the presence of amastigotes.

In addition to 46 healthy individuals in the control group were matched for age, gender, and race. who lived in Al-Baha and were selected from the healthy subjects attending outpatient clinics coming for routine checkups. We estimated the sample size for the study using the formula described by Charan and Biswas [15], with a 1:1 ratio of cases to controls, a confidence level of 95%, a power of 80%, and an estimated prevalence of cutaneous leishmaniasis 0 .02% according to Zaki et al. [16].

The inclusion criteria for cases included Saudi patients, age range from 5–60 years, from Al-Baha region (same dietary habits, and same sunlight exposure) with confirmed CL, and both genders who agreed to participate in this study.

The inclusion criteria for controls include Saudi healthy age, gender, and race-matched individuals who lived in Al-Baha with no evidence of cutaneous leishmaniasis and at least one member of the family with a history of CL to ensure the same risk of vector exposure.

**Fig 1:**
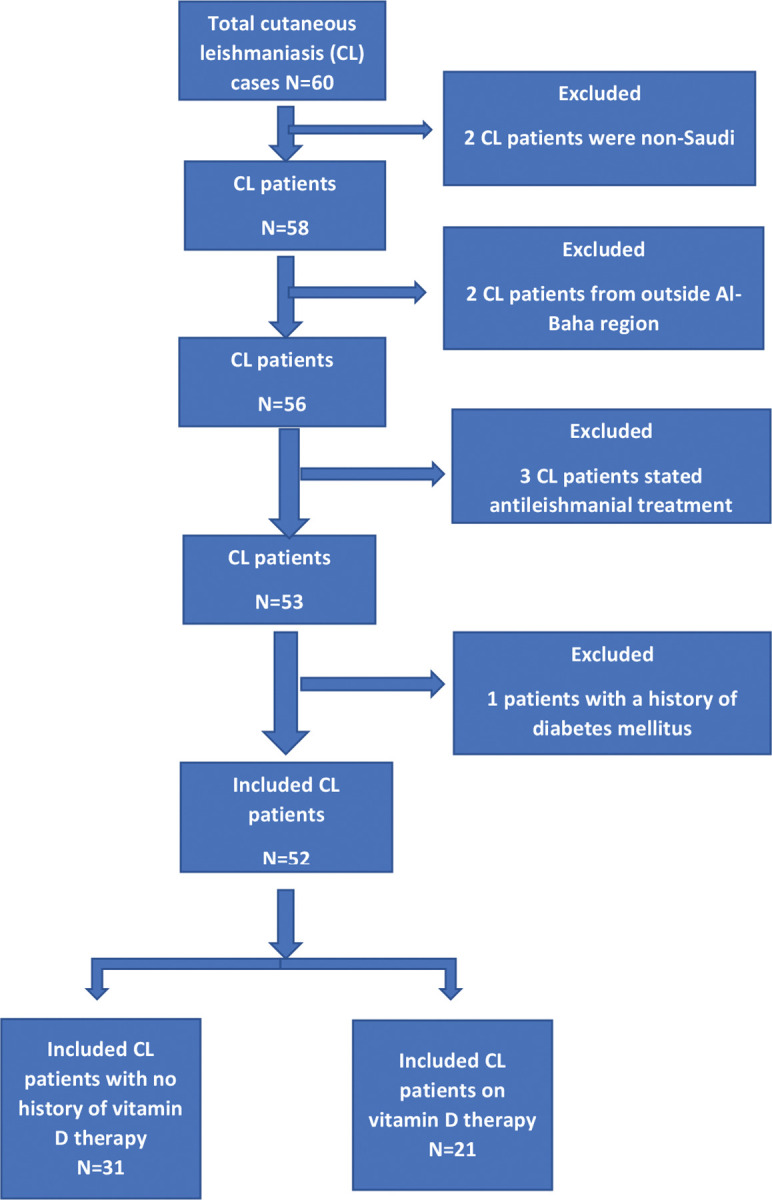
Flow chart of inclusion and exclusion criteria.

Exclusion criteria for both included none Saudi, age below 5 years or above 60 years, living outside Al-Baha, having a history of chronic diseases affecting vitamin D levels like autoimmune disease, diabetes, cancer, and allergies, chronic kidney disease, or a history of allergy to cholecalciferol, history of drugs affecting vitamin D metabolism were excluded from this study as shown in the flow chart (Fig 1).

All participants were subjected to history taking including sunlight exposure, dietary intake of vitamin D, and family history of CL. A clinical examination was performed on each participant to determine the location, size, kind, and number of skin lesions. Patients with CL were treated with intralesional sodium stibogluconate (SSG) 0.5 mL/cm2 of the lesion, twice a week for five to seven injections, depending on the lesion and its response to treatment [17]. For patients with evidence of vitamin D deficiency (n = 21), oral cholecalciferol was administered at a dose of 1500 IU/day if the serum 25-OH vitamin D levels were < 20 ng/mL or 50,000 IU initial loading dose weekly for 8 weeks for severe vitamin D deficient cases (< 10 ng/mL), followed by a maintenance dose at 1000 IU/day to achieve and maintain serum levels greater than 30 ng per mL [18].

### Laboratory evaluation

#### Sampling

From all participants a total of 10 ml of whole blood was collected, 5 ml was transferred to a plane tube and was allowed to clot for 30 minutes and then centrifuged for 10 minutes at 3,500 rpm to separate serum, it was immediately aliquoted and stored at -20°C until the performance of 25-OH vitamin D. The other 5 ml was transferred to ethylene-diamine-tetra-acetic acid (EDTA)-K3 tube for DNA extraction using the spin column technique.

#### Determination of plasma 25-hydroxyvitamin D Levels

Total plasma 25-hydroxyvitamin D (25-OH vitamin D) was measured for all study participants by ELISA technique. The protocol was followed according to the manufacturer’s instructions (Immunodiagnostic Systems Ltd (Boldon, United Kingdom). The intra-assay and inter-assay coefficients of variation were within 10%.

The results were categorized by the Endocrine Society, with levels > 30 ng/ml graded as vitamin D sufficiency, between 20 ng/mL and 30 ng/mL defined as insufficiency, and < 20 ng/mL as deficiency [19].

#### Genotype analysis of VDR gene

This study investigated the polymorphisms of BsmI, ApaI, TaqI, and FokI in the VDR gene.

#### DNA Isolation

Genomic DNA was extracted using the spin column technique (GeneJET Whole Blood Genomic DNA Purification Mini Kit—Thermo Scientific, USA, Cat. no #K0781). The extracted DNA was quantified by measuring the absorbance at 260/280 nm using NanoDrop 2000 spectrophotometer. The DNA concentration of the samples was between 20 and 65 ng/μL. A 260/A 280 ratio represents the purity of the extracted DNA (~1.8 is generally accepted).

#### Genotyping

Genotyping of VDR was done by restriction fragment length polymorphism analysis. The following primer sequences were used.

#### Primer Sequences

- Primer 1: 5′-CAACCAAGACTACAAGTACCGCGTCAGTGA-3′ [20].
- Primer 2: 5′-AACCAGCGGGAAGAGGTCAAGGG-3′ [20].
- Primer 3: 5′-CAGAGCATGGACAGGGAGCAA-3′ [21].
- Primer 4: 5′-GCAACTCCTCATGGCTGAGGTCTC-3′ [21].
- Primer 5: 5′-AGCTGGCCCTGGCACTGACTCTTGCTCT-3′ [20].
- Primer 6: 5′-ATGGAAACACCTTGCTTCTTCTCCCTC-3′ [20].

The restriction fragment length polymorphisms (RFLPs) were coded as Bb, Aa, or Tt, Ff for FokI, BsmI, ApaI, and TaqI respectively. Genotypes were determined as follows; AA, Aa, or aa for ApaI polymorphism; BB, Bb, bb for BsmI polymorphism. TT, Tt or tt for TaqI polymorphism; and FF, Ff or ff for FokI polymorphism. The allele digested by the restriction enzyme was denoted by a lower letter, whereas that not digested was indicated by a capital letter.

Genomic DNA was amplified using a thermal cycler (Biometra, distributed by LABREPCO, Horsham, Pennsylvania). PCR reactions (25 μl) were optimized and contained the following: PCR master mix (12.5 μL). Primer 1 originating in exon 7, and primer 2 originating in intron 8 for the BsmI restriction site (1 μL of 10 pmol/μL each) or primer 3 originating in intron 8 and primer 4 originating in exon 9 for ApaI and TaqI (2 μL of 10 pmol/μL each), primer 5 and primer 6 for detection of the Fok-I restriction site. 5 μL extracted DNA template (10 ng/μL) and 5.5 μL or 3.5 μL double distilled water.

After an initial 5 minutes at 95°C for initial denaturation, DNA was amplified by 35 cycles of 30 seconds at 95°C, then annealing one minute at 64°C (for BsmI), 61°C (for FokI), 60°C (for ApaI and TaqI) and extension at 72°C for 30 seconds (for BsmI, ApaI, and TaqI), 1 minute (for FokI), and a final extension step of 10 minutes at 72°C.

BsmI amplified fragments were 850 base pairs (bp) long was digested with 1 U of BsmI restriction enzyme (FastDigest BsmI by New England Biolabs, Cat. no: R0134S) for 20 minutes at 65°C. Digested DNA fragments were separated by size on a 2% agarose gel and were visualized under short-wave UV light. The 650-bp and 200-bp fragments were produced when the BsmI restriction site was present on both alleles (designated a bb), whereas only one undigested 850-bp fragment was produced when the restriction site was absent (BB).

ApaI amplified fragments were 740 bp long and were digested with 1 U of ApaI restriction enzyme (FastDigest ApaI by Thermo Scientific, USA, Cat. no: #FD1414) at 37°C for 20 minutes. Digested DNA fragments were separated by size on a 2% agarose gel and were visualized under short-wave UV light. The genotype designated as AA in the absence of ApaI site yielding only one fragment of 740 bp, identified as aa in the presence of restriction site yielding two fragments of 515 bp and 225 bp, and heterozygous Aa.

TaqI amplified fragments were digested with 1 U use of 1 U of TaqI restriction enzyme (FastDigest TaqI by Thermo Scientific, USA, Cat. no: #FD0674) at 65°C for 10 minutes. Digested DNA fragments were separated by size on a 2% agarose gel and were visualized under short-wave UV light. The TaqI digestion revealed one obligatory restriction site, the homozygous TT (absence of the specific TaqI restriction site) yielding fragments of 495 bp and 245 bp. The homozygous tt exhibited 290, 245, and 205 bp fragments, and the heterozygous Tt provided 495, 290, 245, and 205 bp fragments.

**Fig 2:**
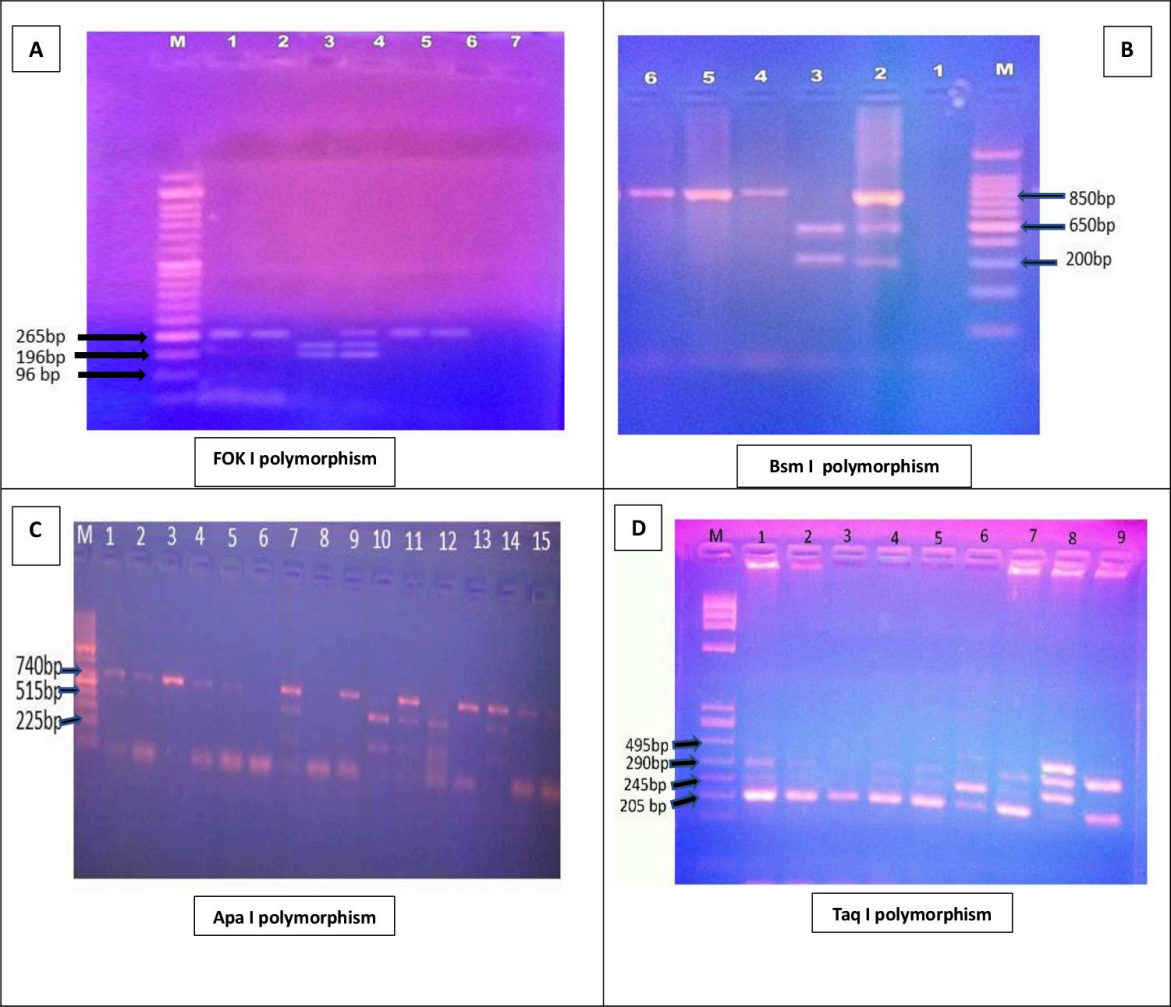
Agarose gel electrophoresis Representative RFLP pattern to distinguish Bsm I, FOK I, Apa I, Taq I polymorphism. A. FOK I polymorphism: Lane 1 DNA ladder and lanes 2–7 patients^’^ samples, the TT (495 bp and 245 bp), Tt (495 bp, 290 bp, 245 bp, and 205 bp) and tt (290 bp, 245 bp, and 205 bp) genotypes. B. Bsm I polymorphism: Lane 1 DNA ladder and lanes 2–8 patients^’^ samples, BB (850 bp), Bb (850, 650, 200 bp), and bb (650, 200 bp) alleles. C. Apa I polymorphism: Lane 1 DNA ladder and lanes 2–16 patients^’^ samples the AA (740 bp), Aa (740 bp, 515 bp, and 225 bp), and aa (515 bp and 225 bp) genotypes. D. Taq I polymorphism: Lane 1 DNA ladder and lanes 2–10 patients^’^ samples the TT (495 bp and 245 bp), Tt (495 bp, 290 bp, 245 bp, and 205 bp) and tt (290 bp, 245 bp, and 205 bp) genotypes.

For the Fok-I The same steps were carried out, amplified fragments were 265 bp were digested with 1 U of FOK-I restriction enzyme (FastDigest FokI by Thermo Scientific, USA, Cat. no: #FD2144) at 37°C for 10 minutes and inactivated the enzyme by heating for 5 min at 65°C, Digested DNA fragments were separated by size on a 2% agarose gel and were visualized under short-wave UV light. In the absence of a FOK-I restriction site, the homozygous FF yielded one band at 265 bp, While the homozygous ff genotype yielded 196 and 69 bp bands; and heterozygote Ff displayed three bands of 265, 196, and 69 bp as shown in Fig 2.

#### Slit skin smear

To prepare slit skin smears, a slot was cut down the active edge of the lesion. Using a scalpel blade with the blunt side, the material was scraped from the gaped walls of the nick. The scabs should be removed from any lesion that had a scab, and tissue material should be scraped from the base to prepare smears. To obtain a smear from fine-needle aspiration (FNA), we used a 23 G needle fixed to a 2ml syringe and applied suction to the lesion edge while rotating the rotator backward and forward.

The slides were then allowed to air-dry before they were fixed in methanol and then stained with Giemsa for detection of amastigotes under the light microscope with an oil immersion lens. Cross-checking by a parasitologist was carried out as a quality control measure. Ridley parasitic index was used to quantify the organisms [22].

#### Skin biopsy

Under local anesthesia and aseptic conditions, two punch biopsies were taken from the lesion measuring 2–3 mm. To prepare a routine histopathological specimen, the biopsy specimen was fixed in 10% formalin, then air-dried, fixed in methanol, and stained with Hematoxylin and Eosin. When Hematoxylin and Eosin failed to detect the organism under the light microscope, Giemsa stain was used.

#### Statistical analysis

Data were analyzed using the Statistical Package of Social Sciences (SPSS) version 20 for Windows (SPSS, Inc., Chicago, IL, USA). For quantitative values, mean and Standard Deviation (SD) were used, while for qualitative values, the number of cases (percentage) was used. The Kolmogorov-Smirnov test was used to determine the distribution of the tested variables. An independent samples t-test was used to assess the significance of differences between continuous variables. For comparing qualitative variables, either the Chi-square or Fisher exact test was utilized, depending on the situation. The DeFinetti program was used to examine the genotypic and allelic illness association studies and the SNPs were tested for Hardy-Weinberg equilibrium. Gene counts were used to assess the genotype and polymorphism frequencies.

The relationship between various Vitamin D genotypes and serum levels of 25-OH vitamin D was evaluated by analysis of variance (ANOVA). ANOVA post hoc test using LSD was used to determine where differences truly came from. P values < 0.05 were considered significant.

## Results

### Epidemiological and clinical data of studied participants

**Fig 3:**
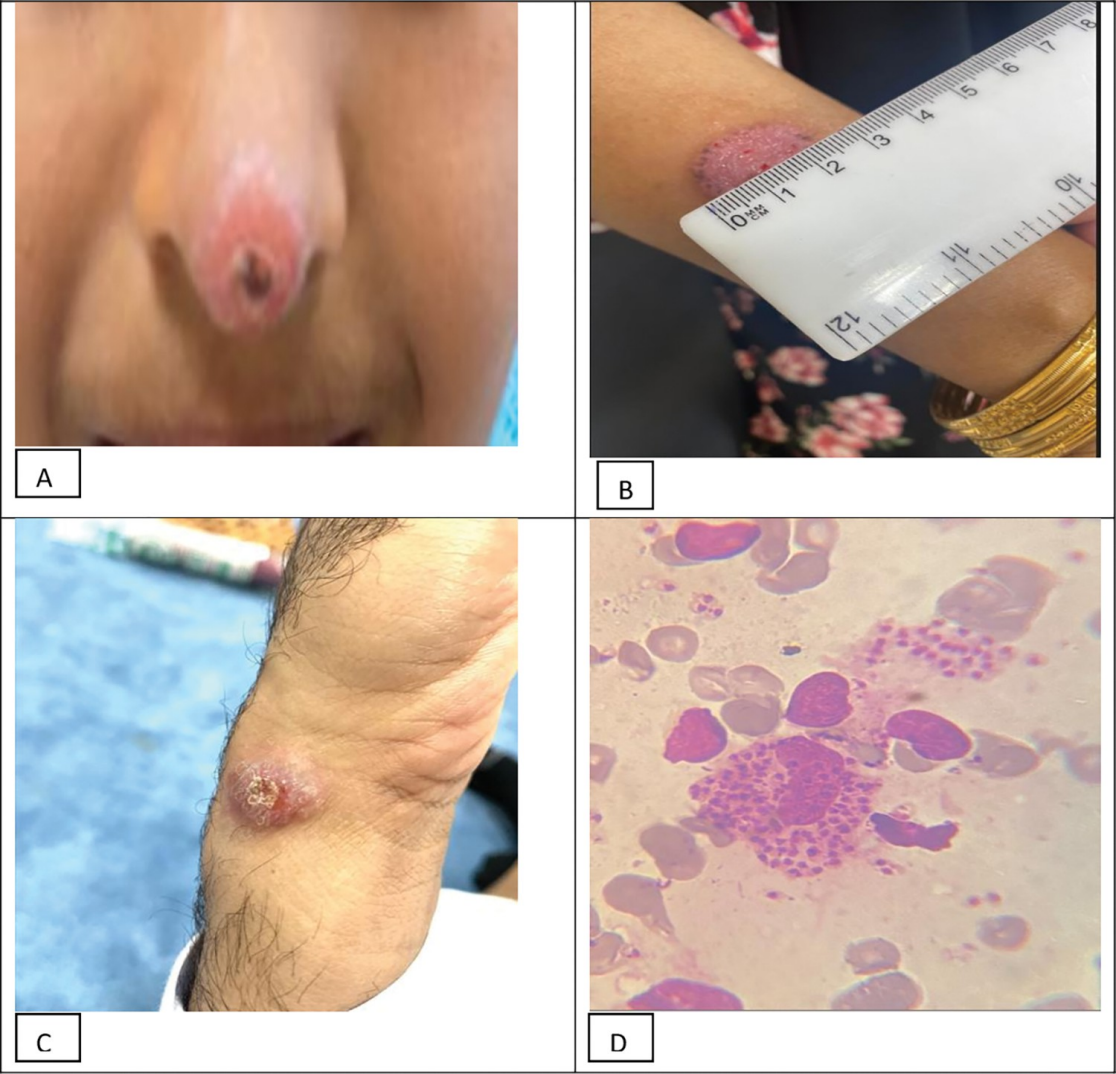
Clinical and skin smear spectrum of cutaneous leishmaniasis. A-Papular cutaneous lesion on the tip of the nose for a 10-year-old patient. B-Ulcerated plaque measuring 2.2cm in diameter on the upper limb, in a 33-year-old female patient. C- Crusted nodular lesion on the upper limb in a 29-year-old male patient. D. Skin smear lesion stained with Giemsa stain demonstrating amastigotes inside and outside the macrophages.

This study evaluated fifty-two patients with CL (31 patients with no history of vitamin D therapy and 21 patients on vitamin D therapy). The mean age of the studied patients was 34.52 ±8.53 years in CL patients without a history of vitamin D therapy and 34.9±6.17 years in CL patients on vitamin D therapy, with age, gender, residency, and race-matched controls (Table 1). Clinical data of the studied patients revealed that the upper limbs account for the majority of cutaneous lesions in 31 patients (59.6%), the face accounted for 14 patients (26.9%), followed by lower limbs in 7 cases (13.5%). The frequency of most frequent lesions was papule (44.2%), nodule (38.4%), and an ulcer (17.3%). Twenty-seven patients presented with a single lesion (51.9%), 17 patients (32.7%) with two lesions, and 8 cases (15.4%) with three lesions. The lesions’ average size was 2.8 ±1.01 mm (range from 1.3–4.7) (Fig 3). The observed lesions lasted from one to thirteen months.

**Table 1:** Epidemiological and clinical data of the studied participants.

Parameters	CL patients with no history of vitamin D therapy N = 31	CL on vitamin D therapy N = 21	Controls N = 46	p
**Age (years)**	34.52 ±8.53	34.9±6.17	37.15± 5.71	0.08
**Gender**				
**Males N (%)**	14 (45.2%)	12 (57.1%)	25 (54.3%)	0.64
**Females N (%)**	17 (54.8%)	9 (42.9%)	21 (45.7%)	
**Residence**				
**Urban N (%)**	23 (74.2%)	13 (61.9%)	27 (58.7%)	0.37
**Rural N (%)**	8 (25.8%)	8 (38.1%)	19 (41.3%)	
**The mean size of the lesions**	**2.77±1.01**	2.18**±0.48**	-	**0.02**
**RPI**	**2.77±1.26**	**2.1±0.77**	-	**0.03**

N, number; CL, cutaneous leishmaniasis; categorical variables are shown as numbers (percentage) and compared by chi-square test. Continuous variables are shown as mean± SD and compared by independent sample t- test between the two groups or ANOVA for the comparison of three groups. p< 0.05 was considered significant.

**Fig 4:**
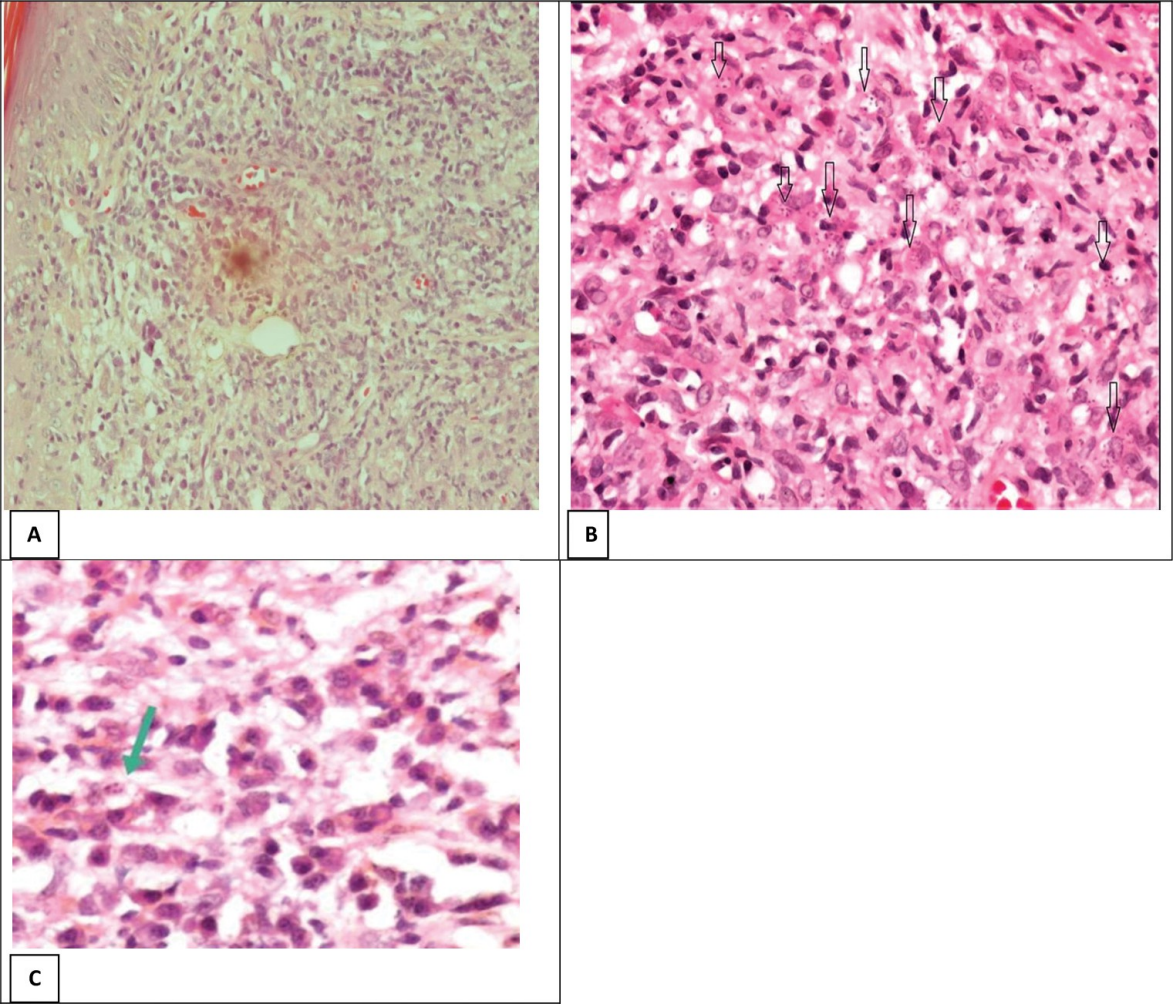
Histopathology of cutaneous leishmaniasis (H&E stain). A- The histopathological changes seen in the epidermis of the CL skin lesion, there are mixed inflammatory cell infiltrates including plasma cells, lymphocytes, and macrophages with intracellular Leishman-Donovan bodies (100x). B- The histopathology sections of the skin biopsy with a high Ridley parasitic index in CL “white arrows” (+4 Ridley parasitic index) (200x). C- The histopathology of CL lesion with low Ridley parasitic index in a CL patient with a history of vitamin D therapy “green arrow” (+2 Ridley parasitic index) (200x).

There was a significantly lower size of lesions and RPI among CL patients on vitamin D therapy compared to CL patients with no history of vitamin D therapy (p = 0.02, 0.03 respectively) (Table 1 and Fig 4).

### Vitamin D levels and categories among studied cases

Serum 25-OH vitamin D levels was significantly lower in patients with CL patients with no history of vitamin D therapy compared to CL patients on vitamin D therapy and controls (22.87±6.4 VS 31.38±3.02, and 32.04±5.1 ng/mL, p = 0.03, 0.006 respectively). When vitamin D categories were compared, it was shown that vitamin D deficiency was significantly higher among CL patients with no history of vitamin D therapy compared to CL patients on vitamin D therapy and controls (p<0.001, 0.002 respectively). However, CL patients on vitamin D therapy had a little non-significantly higher frequency of vitamin D insufficiency compared to CL patients with no history of vitamin D therapy and controls (p > 0.05) (Table 2 and S1 Fig).

**Table 2:** Vitamin D levels and categories among the studied participants.

	CL patients with no history of vitamin D therapy N = 31	CL patients on vitamin D therapy N = 21	Controls N = 46	p
**25- OH vitamin D level ng/mL**	22.87±6.4	31.38±3.02	32.04± 5.1	**P1< 0.001** **P2< 0.001** P3 = 0.51
**Vitamin D levels categories N (%)**				
**Vitamin D sufficiency (≥ 30 ng/mL)**	8 (25.8%)	15 (71.4%)	28 (60.9%)	**P1 = 0.001** **P2 = 0.003** P3 = 0.41
**Vitamin D insufficiency (20–29.9 ng/mL)**	7 (22.6%)	6 (28.6)	10 (21.7%)	P1 = 0.63 P2 = 0.93 P3 = 0.54
**Vitamin D deficiency (<20 ng/mL) N (%)**	16 (51.6%)	0 (0%)	8 (17.4%)	**P1 = <0.0001** **P2 = 0.002** **P3 = 0.04**

N, number; CL, cutaneous leishmaniasis; categorical variables are shown as number (percentage) and compared by chi-square test and continuous data are shown as Mean± SD and compared using ANOVA test to compare mean differences between three groups with LSD post hoc test.

P1 between CL patients without vitamin D therapy and CL patient on vitamin D therapy; p2 between CL patients without vitamin D therapy and controls, P3 between CL patients on vitamin D therapy and controls. p< 0.05 was considered significant.

### Genotypes, and allele frequencies of vitamin D receptor gene polymorphisms

There were no statistically significant differences in BsmI, TaqI, and FokI genotypes or allele frequencies of VDR gene polymorphisms between CL patients and controls (p > 0.05) indicating that the risk of infection may not be affected by these mutations. However, an analysis of ApaI of VDR gene polymorphisms revealed that the frequency of “aa” genotype polymorphisms was significantly lower in CL patients compared to controls (30.7% vs 54.3%, OR = 0.31,95% CI = 0.13–0.72, p = 0.006), and ApaI “a” allele frequency was significantly lower among CL patients compared to controls (30.8% vs 52.2%, p = 0.03) suggesting that this variation may have a protective effect against CL infection. However, the ApaI “A” allele frequency was significantly higher among CL patients compared to controls (69.2% vs 47.8%, OR = 2.45, 95% CI = 1.1–5.6 p = 0.03) implying that this mutation may contribute to the vulnerability to CL infection (Table 3 and S2 Fig).

**Table 3:** Genotypes and allele frequencies for vitamin D receptor (VDR) BsmI, ApaI, TaqI, and FokI in patients with cutaneous leishmaniasis and control subjects.

Genotype/allele	Frequency N (%)		OR	95% CI	p
	CL Patients N = 52	Controls N = 46			
**BsmI genotypes**					
BB	6 (11.5)	4 (8.7)	1.37	0.36–5.19	0.65
Bb	27 (51.9)	27 (58.7)	0.76	0.34–1.69	0.5
bb	19 (36.5)	15 (32.6)	1.19	0.52–2.74	0.68
**BsmI alleles**					
B	42 (80.8)	31 (67.4)	2.03	0.81–5.13	0.13
b	10 (19.2)	15 (32.6)	0.49	0.2–1.2	0.13
**ApaI genotypes**					
AA	13 (32.7)	6 (13)	2.22	0.77–6.43	0.14
Aa	25 (57.7)	15 (32.6)	1.3	0.6–3.1	0.12
aa	14 (30.7)	25 (54.3)	0.31	0.13–0.72	**0.006**
**ApaI alleles**					
A	36 (69.2)	22 (47.8)	2.45	1.1–5.6	**0.03**
a	16 (30.8)	24 (52.2)	0.41	0.18–0.93	**0.03**
**TaqI genotypes**					
TT	8 (15.4)	14 (30.4)	0.42	0.16–1.1	0.08
Tt	26 (50)	18 (39.1)	1.56	0.7–3.48	0.28
tt	18 (34.6)	14 (30.4)	1.2	0.52–2.83	0.66
**TaqI alleles**					
T	32 (61.5)	30 (65.2)	0.85	0.37–1.95	0. 71
t	20 (38.5)	16 (34.8)	1.17	0.51–2.67	0.71
**FokI genotypes**					
FF	9 (17.3)	6 (13)	1.4	0.46–4.3	0.56
Ff	25 (48.1)	25 (54.3)	0.78	0.35–1.72	0.54
ff	18 (34.6)	15 (32.6)	0.74	0.31–1.78	0.50
**FokI alleles**					
F	32 (61.5)	29 (63)	0.83	0.36–1.9	0.66
f	20 (38.5)	17 (37)	1.1	0.47–2.42	0.88

OR, Odds ratio; CI, confidence interval; N, number; CL, cutaneous leishmaniasis; categorical variables are shown as number (percentage) and compared by chi-square test. p< 0.05 was considered significant.

### The haplotype frequencies of the vitamin D receptor gene polymorphisms

A possible haplotypic effect of the studied VDR SNPs on the susceptibility of CL infection was tested and the frequency of seven haplotypes of *Bsm1*, *Apa1*, *Taq1*, *and Fok1 polymorphism* was shown in Table 4. The frequency of the “B-A-T-F” haplotype of VDR gene polymorphisms was significantly more common in CL patients compared to controls (53.8% VS 32.6%, OR = 2.4, 95%, CI = 1.1–5.5, p = 0.04) demonstrating that this mutation may influence the susceptibility to CL infection. On the other hand, the frequency of the “B-a-T-F” haplotype was significantly lower in CL patients compared to controls, and the odds ratio was significantly lower in CL patients compared to controls (OR = 0.24; 95% CI, 0.1–0.7; p = 0.01) indicating that this variation may have a protective effect against CL infection (Table 4).

**Table 4:** The haplotype frequencies of the studied four vitamin D receptor gene polymorphisms in patients with cutaneous leishmaniasis (CL) and controls.

BsmI	ApaI	TaqI	FokI	CL Patients N = 52	Control N = 46	OR (95% CI)	P
B	A	T	F	28 (53.8)	15 (32.6)	2.4(1.1–5.5)	**0.04**
B	A	t	F	5 (9.6)	3 (6.5)	1.52(0.34–6.77)	0.58
B	a	T	F	5 (9.6)	14 (30.4)	0.24(0.1–0.7)	**0.01**
b	A	T	F	1 (1.9)	1 (2.20)	0.88 (0.05–14.5)	0.93
b	A	t	F	6 (11.5)	2 (6.5)	2.9(0.55–15)	0.21
b	a	t	F	6 (11.5)	7 (15.2)	0.7(0.23–2.34)	0.59
b	a	T	F	1 (1.9)	4 (8.7)	0.21(0.02–1.9)	0.16

OR, Odds ratio; CI, confidence interval; N, number; CL, cutaneous leishmaniasis; categorical variables are shown as number (percentage) and compared by chi-square test. p< 0.05 was considered significant.

### The relationship between vitamin D levels, VDR gene polymorphism, lesion size, and parasite load in histopathological specimens

There was no significant association between BsmI, TaqI, and FokI genotypes of VDR gene polymorphisms and 25-OH vitamin D levels (p > 0.05) in CL patients with no history of vitamin D therapy. However, the “Aa” genotype of ApaI VDR gene polymorphisms showed significantly higher 25-OH vitamin D levels compared to “|AA” and “aa” genotypes polymorphisms (p = 0.02), and the post hoc analysis revealed significantly lower levels of 25-OH vitamin D concentration of “Aa” compared to “aa” (p = 0.009) (Table 5 and S3 Fig).

Additionally, there was no significant association between all vitamin D receptor genotype frequencies and Ridley parasitic load index (RPI) except for ApaI (p = 0.02) in CL patients with no history of vitamin D therapy. Post hoc analysis showed a significantly higher RPI in ApaI “Aa” genotype compared to the” aa” and “AA” of the ApaI genotype polymorphism (p = 0.02, 0.022 respectively) (Table 6).

**Table 5:** Serum levels of vitamin D according to vitamin D receptor genotypes among patients with cutaneous leishmaniasis with no history of vitamin D therapy.

Genotypes	25-(OH) D levels	p
**BsmI genotypes**		
BB	35.5±14.2	0.17
Bb	21.8±4.2	
bb	20.2±3.2	
**ApaI genotypes**		
AA	19±3.37	0.02*
Aa	17.67±2.84	
aa	37.86±8.56	
**TaqI genotypes**		
TT	27±10.5	0.79
Tt	22±3.9	
tt	21.7±4.1	
**FokI genotypes**		
FF	26.3±8.8	0.77
Ff	23±4.3	
ff	22.9±2.9	

LSD* between AA and aa (p = 0.01), Aa and aa (p = 0.009), continuous variables are shown as mean± SD and compared by ANOVA to compare means with LSD post hoc test. p< 0.05 was considered significant.

**Table 6:** The Ridley parasitic load index (RPI) according to vitamin D receptor genotypes among patients with cutaneous leishmaniasis with no history of vitamin D therapy.

Genotypes	RPI	p
**BsmI genotypes**		
BB	2.8±0.95	0.54
Bb	3.1±1.1	
bb	2.5±1.4	
**ApaI genotypes**		
AA	2.2±0.9	0.02*
Aa	3.4±1.2	
aa	2.1±1.2	
**TaqI genotypes**		
TT	3.3±1.2	0.07
Tt	3.1±1	
tt	2.1±1.1	
**FokI genotypes**		
FF	3.1±1.1	0.28
Ff	3±1.2	
ff	2.8±1.2	

LSD between Aa and aa and between Aa and AA (p = 0.020 and 0.022 respectively), continuous variables are shown as mean± SD and compared by ANOVA to compare means with LSD post hoc test. p< 0.05 was considered significant.

**Fig 5:**
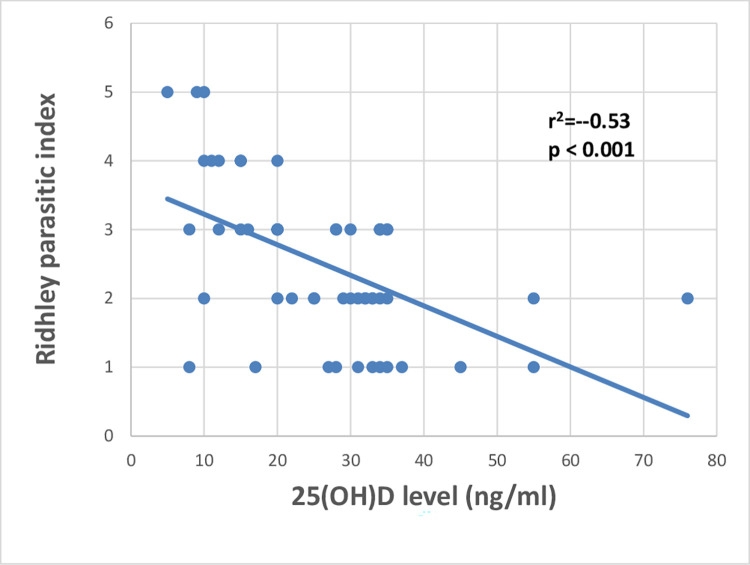
Showed a highly significant negative correlation between Ridhley parasitic index and vitamin D levels (r62 = -0.53, p < 0.001).

**Fig 6:**
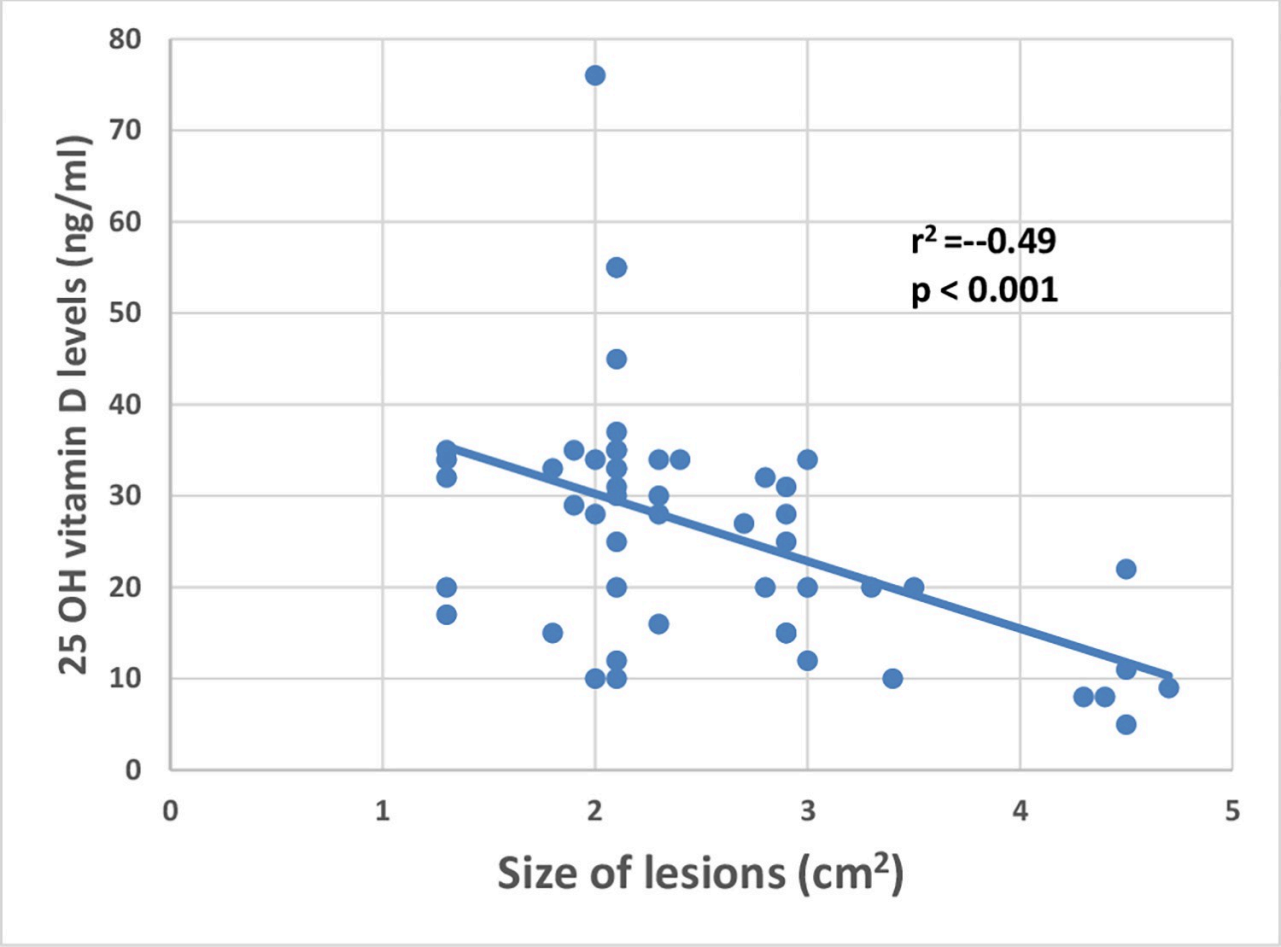
Showed a highly significant negative correlation between the size of the lesions and vitamin D levels (r2 = -0.49, p < 0.001).

After that, we evaluated the possible association between vitamin D levels, Ridley parasitic load index, and size of the lesions and we found that RPI and size of the lesions showed s significant negative correlation with vitamin D levels (r^2^ = -0.53, p < 0.001; r^2^ = -0.49, p < 0.001 respectively) (Figs 5 and 6).

## Discussion

The most common form of leishmaniasis is cutaneous leishmaniasis (CL) and it is an important neglected tropical skin disease with significant public health implications. In Saudi Arabia, over 19.000 cases of CL have been documented over the last 7 years. It is caused by a group of protozoan intracellular parasites belonging to the genus *Leishmania* and transmitted through the bite of blood-sucking female sandfly species [23].

A remarkable lack of data exists on the associations between CL, 25-OH vitamin D levels, and VDR gene polymorphisms. Moreover, no data was recorded about the association between the parasite load and vitamin D gene polymorphism in humans. Therefore, this study aimed to investigate the relationship between vitamin D status, VDR gene polymorphisms (BsmI, ApaI, TaqI, and FokI), and VDR haplotype with parasite tissue load and susceptibility to CL.

In the present study, despite all the participants living in Al-Baha with the same traditional or religious dress, with the same environmental conditions including sunlight exposure, the frequency of vitamin D deficiency was significantly higher in CL patients who were not on vitamin D therapy compared to CL patients who were on vitamin D therapy and controls and the 25-OH vitamin D levels were significantly lower in CL patients who were not on vitamin D therapy compared to CL patients on vitamin D therapy and controls.

Our results were in line with an experimental study on canine leishmaniasis by Rodriguez-Cortes et al, who reported that vitamin D levels in dogs with leishmaniasis were significantly lower than in non-infected and asymptomatic dogs [24]. However, some human research reported no statistically significant difference in 25-OH vitamin D levels between CL patients and controls [2,25,26].

In the current work, the significantly high frequency of vitamin D deficiency in CL patients who were not on vitamin D therapy could be a result of the disease as well as a contributing factor to the disease’s development due to its immunomodulatory effect. Vitamin D deficiency impairs both the innate and adaptive immune responses, decreased expression of genes encoding antimicrobial peptides including cathelicidin production which may influence susceptibility to infection and parasite clearance, a more pro-inflammatory state, and decreased autophagy, which is critical for the control of intracellular pathogens. Therefore, our results strongly support the concept that vitamin D deficiency may enhance susceptibility to CL and is emerged as a risk factor for intracellular pathogens infections including *Leishmania* species [27].

The varying vitamin D deficiency rates in CL patients among different studies may be related to a diversity of contributing factors, such as environmental factors (sun exposure, dietary supplements), sample size, age of participants, race, skin type, BMI, and genetic background of the participants. Also, variants in gene loci affecting cholesterol synthesis, hydroxylation, and vitamin D transport are known to affect vitamin D status [28]. The increased frequency of vitamin D deficiency among our patients may result from limited exposure to sunshine (Al-Baha location at 2,500 meters above sea level), insufficient dietary supplements due to lack of awareness about vitamin D deficiency in Al-Baha region as reported by Abukhelaif et al., [29].

Although vitamin D therapy has been extensively examined in many diseases, little is known about its involvement in parasite disorders such as cutaneous leishmaniasis. However, the benefit of vitamin D supplementation against cutaneous leishmaniasis infection has not yet been demonstrated in humans, and further investigation is needed.

In a murine model, Ramos-Martinez et al. reported that vitamin D treatment can reduce the disease progression in the *Leishmania mexicana*-infected animal mice model. Additionally, vitamin D plays a beneficial effect in *Leishmania infantum* infection in dogs [24,30]. In other protozoal infections, oral vitamin D was found to protect mice from experimental cerebral malaria [31] and vitamin D overload protected Balb/c mice against infection with the CL and Y strains of *Trypanosoma cruzi* [32]. A study in Uganda reinforces this evidence in humans by demonstrating that, with every 1 ng/mL increase in plasma 25-OH vitamin D, the odds of having cerebral malaria declined by 9% [33]. Also, Rajapakse et al., reported that 1,25-dihydroxy vitamin D3 may inhibit intracellular *Toxoplasma gondii* proliferation *in vivo* and *in vitro* [34].

In the present work, we evaluated the association between vitamin D status and CL clinical severity, and parasite load. The size of the lesions and RPI was significantly lower among CL patients on vitamin D therapy compared to CL who were not on vitamin D therapy. In addition, we demonstrated a significant negative correlation between vitamin D levels and the parasite load in histopathological findings; and the size of the lesions which means that the levels of vitamin D could affect the extent of CL. These results reinforce the results of the experimental study on canine leishmaniasis which showed that vitamin D concentrations were strongly negatively correlated with clinical severity, parasite load, and anti-*Leishmania* IgG levels [24]. However, Shabandoust et al. demonstrated no significant relationship between the serum level of vitamin D and the number of lesions in CL [26], and a murine model demonstrated that mice lacking the vitamin D receptor (VDRKO) on a genetically resistant background developed fewer *Leishmania major*-induced lesions and had fewer parasites at the site of infection during the peak of infection, but these findings may not be applied to humans due to different genetic background [35–38]. Therefore, vitamin D had a significant impact on the development of cutaneous lesions in animal models of leishmaniasis where vitamin D-deficient rats acquired lesions [37]. The high parasite load and the large size of the lesions among CL patients with vitamin D deficiency could be explained by the impact of vitamin D on the adaptive immune system, especially on T cells subtypes (TH1 cells, TH2 cells, TH17 cells, and T regulatory cells) [39].

Understanding the host immunogenetics of leishmaniasis may aid in understanding the mechanism underlying disease progression in genetically susceptible individuals. The uncontrolled pro-inflammatory cytokine production appears to contribute to ulceration and lesion severity and many of these proinflammatory cytokines, such as IL-1, IL-6, IL-8, and TNF-α are suppressed with vitamin D therapy. Additionally, vitamin D increases the antimicrobial peptide production and phagocytosis by macrophages and intracellular killing by decreasing the binding of the phagosome with the lysosome and decreasing the production of nitric oxide free radicals to inhibit the growth of intracellular microorganisms [37]. It was proposed that vitamin D and calcium stimulate catenin and adherent junction signaling to aid in the re-epithelialization of wounds and to combat drug-resistant leishmaniasis, an adjuvant therapy-based vitamin D therapy is desperately needed to eradicate *Leishmania* infection [36]. Therefore, maintenance of adequate vitamin D levels in CL patients in addition to currently available anti-leishmanial medications is proving to be a substantial and cost-effective choice in the treatment toolkit for reducing parasite burden, lesion size, and promoting infection clearing.

The interaction of the parasite, host, and environment determines susceptibility to CL and its clinical symptoms. CL susceptibility is influenced by host variables that are genetically defined and immune response variability. VDR genes continue to be significant candidate genes among those involved in immune system regulation. The involvement of vitamin D/VDR in anti-inflammatory action has been demonstrated in numerous infections. Vitamin D receptors also play a role in promoting immune regulation through multiple pathways [40–43]. The integrity of the vitamin D receptor is another crucial component in how well the vitamin D/VDR complex functions; if there are any flaws, it will be difficult for cells to absorb the active form of vitamin D. The gene polymorphism of the vitamin D receptor can be used to determine the quality of the receptor. It’s interesting to note that some microbes can alter the vitamin D/VDR signaling pathways, which reduces the expression of VDR and weakens the innate immune response. [44,45].

The 1,25-dihydroxy vitamin D3 action is mediated by the nuclear transcription factor VDR. It has 11 exons, of which 2–9 exons are actively transcribed, and is found on the long arm of chromosome 12. The VDR gene’s intron 8 has both the ApaI and BsmI sites. The apaI polymorphism negatively regulates the expression of VDR [46].

In the current study, four vitamin D gene genetic variants (BsmI, ApaI, TaqI, and FokI) were examined for associations with susceptibility to CL infection and parasite load in the skin biopsy To determine the potential contribution of the VDR polymorphisms to the susceptibility to infection by Leishmania species. Our study has shown that the “A” allele in ApaI SNP of VDR is more frequent among CL patients compared to controls, in addition, individuals with the “A” allele had a 3.7-fold higher risk for developing CL compared to controls, thus revealing that patients were more susceptible to CL. However, “aa” genotype polymorphisms in ApaI SNP and “a” allele showed a significant trend towards a lower frequency among patients with CL compared to control indicating that this association could represent a protective effect against CL.

Vitamin D is a crucial immune system molecule and its deficiency is a risk factor for a wide range of diseases. To assess any potential correlations between the VDR gene polymorphism, parasite load in skin biopsy, and vitamin D levels among CL patients, the levels of 25-OH vitamin D were also assessed. According to the genotype distribution, patients with ApaI "aa" genotype had significantly higher 25-OH vitamin D levels than those with the "AA" or "Aa" genotypes, and CL patients with the "aa" genotype had significantly lower parasite loads than those with the "AA" and "Aa" genotypes. In addition, CL patients with the “Aa” genotype had significantly the lowest vitamin D levels and highest parasite load compared to AA and aa genotype. These results imply that the parasite burden and 25-OH vitamin D levels among CL patients are related to the ApaI VDR polymorphisms. Our results support that the ApaI SNP of VDR polymorphisms may cause VDR malfunction, which would result in poor vitamin D action. In addition, poor vitamin D action and vitamin D deficiency impair the innate and adaptive immune systems which may enhance vulnerability to CL. As a result, it has been postulated that ApaI gene polymorphism may impact vitamin D responsiveness [47].

Additionally, the frequency of the “B-A-T-F” haplotype was significantly more common in patients with CL compared to controls and is associated with CL, as the odds of having the haplotype were 2.86 times higher in CL individuals compared to controls. However, the frequency of the “B-a-T-F” haplotype of vitamin D receptor gene polymorphisms was significantly greater in control patients compared to CL patients and could be protective against CL, as the odds of having the haplotype were lower among CL patients compared to controls, suggesting that this haplotype may have the potential to protect against cutaneous leishmaniasis susceptibility These results revealed that the presence of the “A” allele in ApaI SNP of VDR and “B-A-T-F” haplotype increases the susceptibility to CL, while “a” allele and “B-a-T-F*”* haplotype may have potential protection against CL and these are novel findings which have not been reported before. A previous study found that the A allele in the ApaI SNP was related to lower serum TNF- levels [48]. TNF-α, IFN-λ, and other proinflammatory cytokines may influence the outcome of leishmaniasis because they play an important role in macrophage activation and activate inducible nitric oxide synthase. The deficiency of TNF signaling reduces the activities of IFN- λ and iNOS, and as a result, the immune system is unable to prevent infection development and the amount of TNF-α in CL lesion biopsies was substantially linked with lesion size in CL [49,50]

The polymorphisms in the VDR gene are known to alter calcium metabolism, which plays aa important role in the feedback mechanism of vitamin D levels, which may account for the relationship of the ApaI genotype with vitamin D levels. It’s also feasible that the ApaI polymorphism is in linkage disequilibrium with another marker, which could impact vitamin D levels.

Furthermore, the ApaI VDR polymorphism has been linked to the pathophysiology of other infections. A meta-analysis by Areeshi et al. revealed that the ApaI VDR polymorphism is strongly related to a lower risk of tuberculosis infection [46], and Hoan et al. demonstrated that it is related to clinical outcomes and liver disease progression in Vietnamese HBV-infected patients [51].

In this study, the association between BsmI, TaqI, and FokI polymorphisms in VDR with 25-OH vitamin D and susceptibility to cutaneous Leishmaniasis and the parasite load was not observed. Similar to this result, Shabandoust et al. observed that genotypes of vitamin D receptors did not affect the susceptibility to CL due to *Leishmania*. *tropica* [26].

Rodriguez et al. investigated the relationship between the VDR gene polymorphisms and susceptibility to *T*. *cruzi*, infection and demonstrated that the VDR gene polymorphisms may have an impact on the immune response against *T*. *cruzi*, raising the risk of cardiac problems in those patients [52].

According to research on VDR gene variants in other infectious diseases, FokI has been linked to tuberculosis in populations of various ethnic backgrounds, albeit the findings are conflicting. For instance, a meta-analysis and a case/control investigation revealed that, in Chinese and Iranian patients, respectively, the FokI A allele imparted susceptibility to this illness [43,53]. However, in a Moroccan population, additional research found links between this genotype with pulmonary tuberculosis protection [54]. Leprosy, an infectious condition brought on by another species of Mycobacterium, has also been studied with VDR FokI. In this instance, homozygosity of FokI A was linked to a greater incidence of leprosy in a community of Indians. [44].

The VDR locus has been investigated for its potential link to illness vulnerability, although results have frequently varied among people around the world. This discrepancy might be brought about by interactions between several genetic or environmental factors, racial differences, or both. VDR polymorphism serves as a marker for functional variations that impact VDR expression levels but may not directly cause disease. According to studies, VDR polymorphism has demonstrated that the VDR gene may play a key role in determining the quantity of VDR mRNA, VDR protein, and the ensuing downstream vitamin D-mediated action [55,56].

Overall, these results suggested that the serum level of 25-OH vitamin D, and ApaI of VDR polymorphisms, correlated with parasitic load and could alter the susceptibility to CL, whereas BsmI, FokI, and TaqI polymorphisms did not. Maintenance of adequate vitamin D levels in CL patients aids in reducing parasite load, and burden, and promoting infection clearing.

Despite these findings, more research on a bigger sample size and in different regions of Saudi Arabia is required for a more precise interpretation of these findings. In addition, other gene variations in the vitamin D pathway, as well as epigenetic factors, which can play a role in disease development must be investigated. Future work resulting from this study would involve looking into different dosages and methods of vitamin D administration to determine which is suitable for treatment in such cases.

## Conclusions

These results revealed that the serum level of 25-OH vitamin D, and the polymorphisms in ApaI of vitamin D receptor, correlated with parasitic load and could alter the susceptibility to CL. whereas BsmI, FokI, and TaqI polymorphisms did not. Correction of vitamin D levels may aid in CL management.

## Supporting information

S1 Fig Frequency of 25- OH vitamin D deficiency among cutaneous leishmaniasis (CL) cases with no vitamin D therapy and controls. (TIFF)

S2 Fig Frequency of ApaI allele among cutaneous leishmaniasis (CL) cases and controls. (TIFF)

S3 Fig Association between serum levels of 25-OH and ApaI genotypes of vitamin D receptors among patients with cutaneous leishmaniasis with no history of vitamin D therapy. (TIFF)

S1 Data Supporting data for figures and tables. (XLSX)
