# Supplementary figures and images for: Vitamin D status, vitamin D receptor gene polymorphism, and haplotype in patients with cutaneous leishmaniasis: Correlation with susceptibility and parasite load index
Source: PLoS Negl Trop Dis. 2023 Jun 15;17(6):e0011393. doi: 10.1371/journal.pntd.0011393 (PMC10270342; doi:10.1371/journal.pntd.0011393)

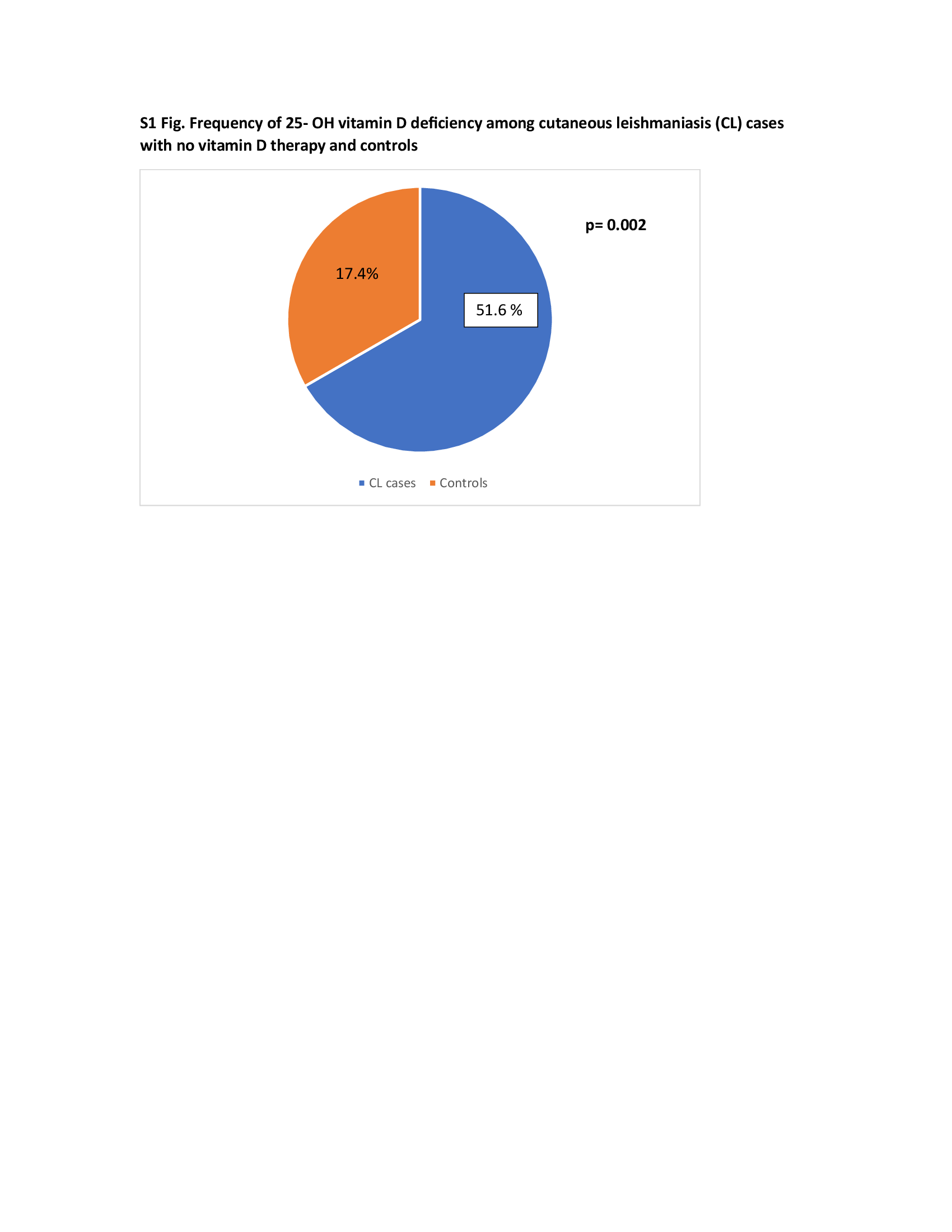

Supplement: S1 Fig — (TIFF) [file pntd.0011393.s001.tiff]

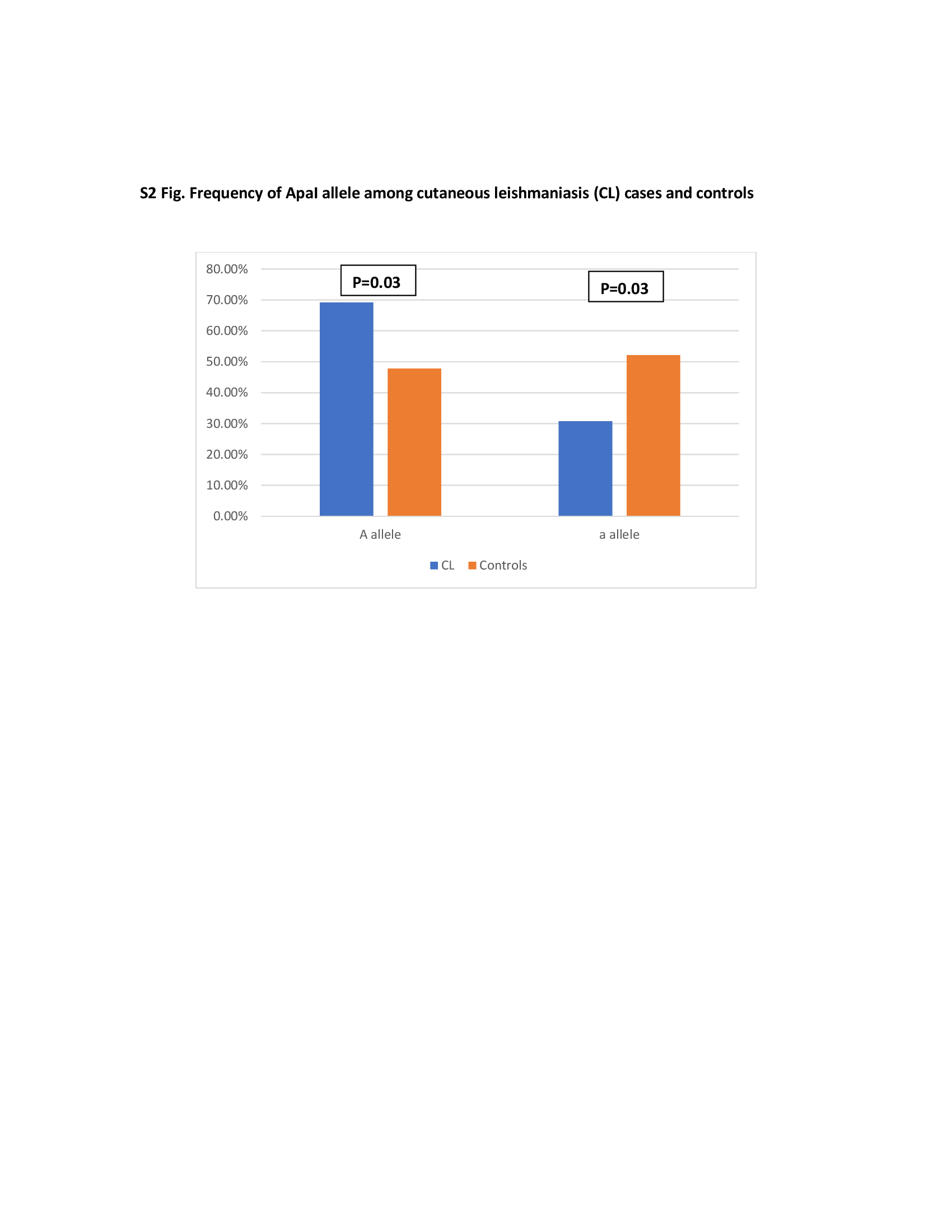

Supplement: S2 Fig — (TIFF) [file pntd.0011393.s002.tiff]

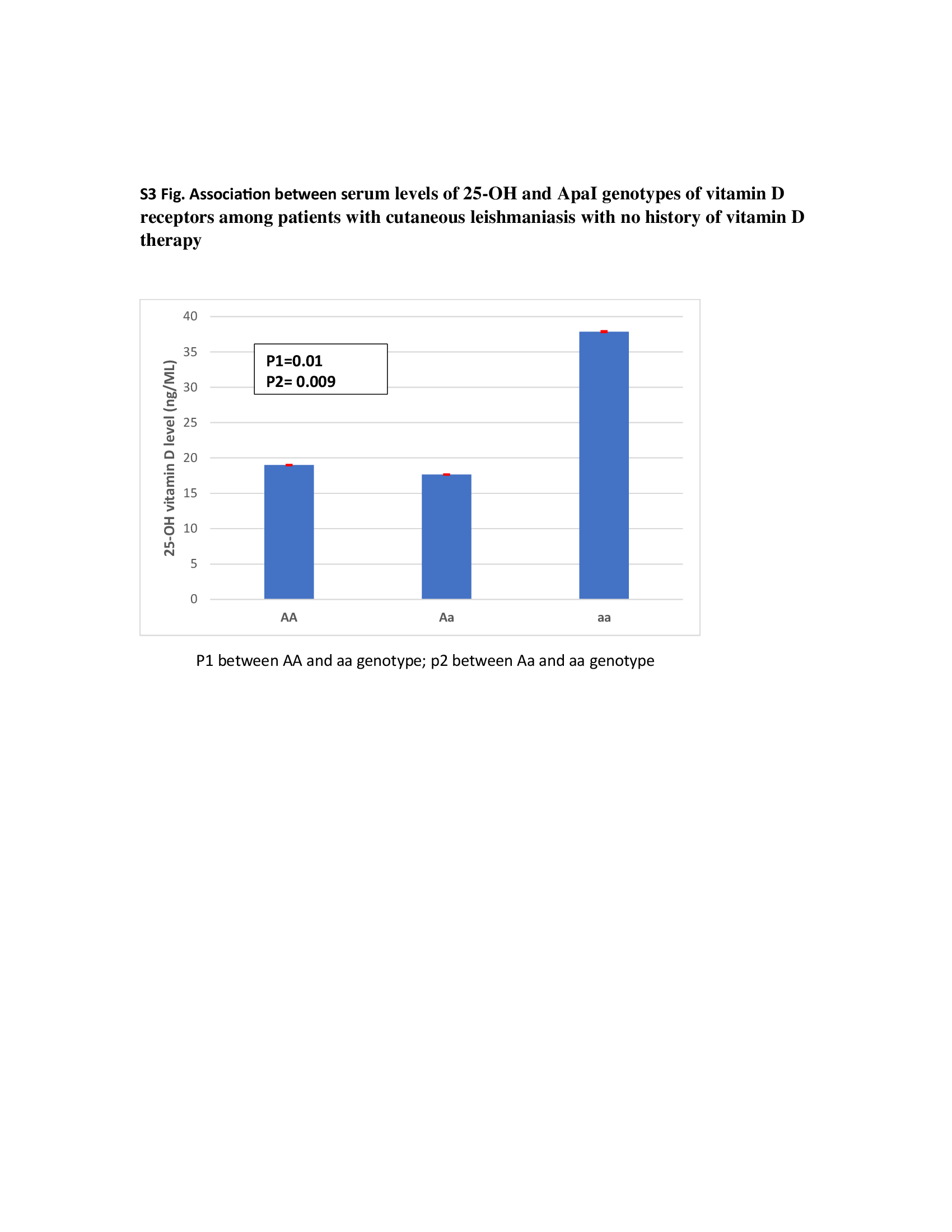

Supplement: S3 Fig — (TIFF) [file pntd.0011393.s003.tiff]
